# Cell signalling pathway regulation by RanBPM: molecular insights and disease implications

**DOI:** 10.1098/rsob.170081

**Published:** 2017-06-28

**Authors:** Louisa M. Salemi, Matthew E. R. Maitland, Christina J. McTavish, Caroline Schild-Poulter

**Affiliations:** Robarts Research Institute, Department of Biochemistry, Schulich School of Medicine and Dentistry, The University of Western Ontario, 1151 Richmond Street North, London, Ontario, CanadaN6A 5B7

**Keywords:** RanBPM, CTLH complex, Gid, cancer, Alzheimer disease

## Abstract

RanBPM (Ran-binding protein M, also called RanBP9) is an evolutionarily conserved, ubiquitous protein which localizes to both nucleus and cytoplasm. RanBPM has been implicated in the regulation of a number of signalling pathways to regulate several cellular processes such as apoptosis, cell adhesion, migration as well as transcription, and plays a critical role during development. In addition, RanBPM has been shown to regulate pathways implicated in cancer and Alzheimer's disease, implying that RanBPM has important functions in both normal and pathological development. While its functions in these processes are still poorly understood, RanBPM has been identified as a component of a large complex, termed the CTLH (C-terminal to LisH) complex. The yeast homologue of this complex functions as an E3 ubiquitin ligase that targets enzymes of the gluconeogenesis pathway. While the CTLH complex E3 ubiquitin ligase activity and substrates still remain to be characterized, the high level of conservation between the complexes in yeast and mammals infers that the CTLH complex could also serve to promote the degradation of specific substrates through ubiquitination, therefore suggesting the possibility that RanBPM's various functions may be mediated through the activity of the CTLH complex.

## 1. Overview

RanBPM (Ran-binding protein microtubule organizing centre, also known as RanBP9) was first described in 1998 and was identified as a 55 kDa protein found to interact with Ran, a guanine triphosphatase (GTPase) involved in nucleocytoplasmic transport, by a yeast two-hybrid screen [1]. RanBPM was found to be localized to the centrosome, hence named Ran-binding protein microtubule organizing centre [1]. However, these findings were later dismissed as it was found that the 55 kDa protein product initially identified was a truncation of a longer 90 kDa protein [2]. The full-size RanBPM protein was able to only weakly associate with Ran and had no role in nucleocytoplasmic transport [2]. New antibodies generated against the full sized RanBPM did not show centrosomal localization and instead displayed staining throughout the whole cell while being concentrated in the nucleus [2]. Over the next 19 years, many studies identified RanBPM as interacting with various proteins, suggesting its involvement in many cellular processes. Although no specific function could be attributed to RanBPM based on its primary structure, some studies demonstrated a biological effect of RanBPM attributed to these interactions, while in other studies a biological function was implied only based on the specific interaction identified. RanBPM's function only started to emerge from studies in yeast, which showed that the RanBPM yeast homologue (Gid1, Vid30) is part of a large E3 ligase complex [3]. Here, we review RanBPM's role in apoptosis, transcription regulation, cell migration, adhesion and morphology and how these pathways contribute to RanBPM's function in cancer, development as well as Alzheimer's disease (AD).

## 2. Domains, conservation and expression

**Figure 1.**
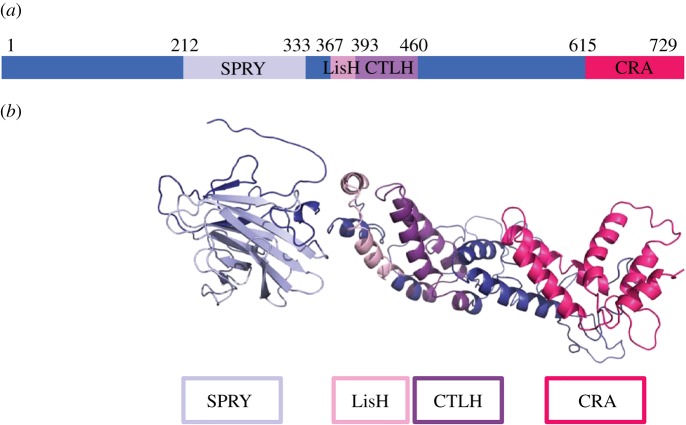
(*a*) Schematic diagram of full-length wild-type RanBPM. The conserved domains are SPRY (Sp1a kinase and ryanodine receptor), LisH (lissencephaly type-1-like homology), CTLH (C-terminal to LisH) and the C terminal CRA (CT-11-RanBPM). Figure adapted from [4,5]. (*b*) Predicted three-dimensional structure of RanBPM. The conserved domains, SPRY, LisH, CTLH and CRA domains, are shown in light-blue, light-pink, violet and hot-pink, respectively. Structure prediction was performed by the RaptorX Structure Prediction server [6–9].

Unlike other Ran-binding proteins, with the exception of RanBP10, RanBPM does not have a Ran-binding domain [1]. However, multiple homology domains have been revealed through sequence analyses that offer potential protein–protein interaction sites to be further examined. RanBPM consists of five potential protein-interaction domains (figure 1*a*). The highly disordered N-terminus of RanBPM is a proline and glutamine-rich region [2] with six prototypical SH3-binding domains [10] predicted to bind Src and Grb2 with high affinity [11]. RanBPM also contains a SPRY (Sp1A kinase and ryanodine receptor) domain [1,12], a structural motif that mediates protein–protein interactions [13,14]. The SPRY domain was first identified in ryanodine receptors and *Dictyostelium discoideum* dual specificity kinase Sp1A [15] and has since been shown to be harboured in a variety of proteins [14]. The majority of SPRY-containing proteins are poorly characterized. However, a great number of human SPRY-containing proteins have been identified as E3 ubiquitin ligases, with most also containing a RING (really interesting new gene) domain [14]. Adjacent to the SPRY domain, RanBPM contains a LisH (lissencephaly type-1-like homology) and a CTLH (C-terminal to LisH) domain. These domains have been found to contribute to the regulation of microtubule binding [16], and to promote homodimerization and oligomerization [17–19]. Lastly, at the C-terminal region of RanBPM, the CRA (CT11-RanBPM) domain is predicted to have six helices reminiscent of the death domain and has been identified as a novel protein–protein interaction domain [13].

The RaptorX Structure Prediction server was used to interpret multiple homologue template structures from previously deposited Protein Data Bank (PDB) files and predict the tertiary structure of RanBPM (figure 1*b*) [6–9]. Various domains of RanBPM lack homologue template structures within PDB, therefore their predicted structure was determined through the incorporation of structural data from evolutionarily distant protein sequences as templates to estimate stable protein-folds. Highly disordered sections of the protein, such as the proline/glutamine-rich N-terminus and the linker region between the CTLH and CRA domain, were either omitted or interpreted as a coil, respectively. Several existing homologous crystal structures of the SPRY domain were employed to estimate approximate folding of the domain with the least entropy. Recent structural studies have revealed that the RanBPM SPRY domain forms a β-sandwich fold, composed of two seven-stranded anti-parallel β-sheets, with two helices located at both the N- and C-termini [20]. The predicted structure shown here does not incorporate the recently elucidated structure of the RanBPM SPRY domain; it does, however, integrate homologue template structures from PDB. The LisH/CTLH domain is depicted to form an anti-parallel helix bundle, probably functioning as the site for protein dimerization and oligomerization. As previously mentioned, the CRA domain is predicted to have a high propensity for α-helices [13]. Visually, the CRA domain was depicted with six α-helices; however, it could be debated that helix I and II may in fact form a single helix, potentially negating its resemblance to the death domain superfamily. RanBPM shares similar domain architecture with its paralogue RanBP10 and they have been shown to associate [21]; however, this review will only focus on RanBPM.

RanBPM is ubiquitously expressed in different tissue types with higher expression observed in the brain, heart, skeletal muscles and testes [22,23]. RanBPM is well conserved in mammals, in fact the mouse and human proteins are over 90% identical and their differences fall within the N-terminus [2]. RanBPM orthologues have also been identified in plants [24]. A RanBPM homologue, vacuolar import and degradation (Vid)30 or glucose-induced degradation (Gid)1, has been identified in yeast [3,25]. Gid1 also contains SPRY, LisH, CTLH and CRA domains [3]. RanBPM has been shown to be part of a large 670 kDa protein complex termed the CTLH complex [2,26,27]. Similarly, Gid1 has been found to be a component of a large protein complex made up of several other Gid proteins, called the Gid complex [3,25,27]. The human homologues of the majority of these proteins have been found to be part of the human CTLH complex [28]. Therefore, RanBPM and most of the CTLH complex members are well conserved in eukaryotic lineages.

RanBPM is localized in both the nucleus and the cytoplasm [2] and has also been found to be present at the plasma membrane as well as within the chromatin fraction [23,29,30]. Work in our laboratory identified a nuclear export signal in the N-terminal region of RanBPM as well as a nuclear localization signal (NLS) in the C-terminal region, which acts secondary to a non-canonical NLS within the first 25 amino acids of RanBPM [4]. We have also found that the SPRY, LisH and CTLH domains are important in cytoplasmic localization or retention, as deletions of these domains resulted in nuclear localization. Interestingly, RanBPM was found to associate with both microtubules and chromatin which may function to retain RanBPM in the cytoplasm and nucleus, respectively [4].

How RanBPM is regulated remains largely unknown, however there is evidence that its function may be modulated by various pathways. RanBPM is deubiquitinated by ubiquitin-specific protease 11 (USP11) and degraded by the ubiquitin–proteasome pathway [31] and RanBPM mRNA is regulated by microRNA-101 [32]. RanBPM has been demonstrated to be phosphorylated after genotoxic stress such as ultraviolet treatment and osmotic shock [29]. Cyclin-dependent kinase 11 p46 fragment (CDK11^p46^) and polo-like kinase 1 (PLK1) phosphorylate RanBPM *in vitro* [33,34]. Although these reports suggest that RanBPM is subjected to a number of post-translational modifications, for the most part, the outcome of these modifications remains unknown. Interestingly, in a recent study, ataxia telangiectasia mutated (ATM) was shown to phosphorylate RanBPM at an ATM consensus sequence following ionizing radiation (IR)-induced DNA damage and RanBPM subcellular localization was reported to change upon IR treatment in an ATM-dependent manner [35,36]. Following IR-induced ATM phosphorylation, RanBPM was found to rapidly relocalize to the nucleus in an ATM-dependent manner, as inhibition of ATM prevented this nuclear accumulation [36]. At later time points, RanBPM was found to relocalize to the cytoplasm [36]. Our earlier studies demonstrated that this cytoplasmic relocalization occurred 24 h after IR and persisted up to 72 h [5]. Palmieri *et al*. also showed that downregulation of RanBPM resulted in reduced ATM acetylation, suggesting that in turn, RanBPM may affect ATM activation [36]. Overall, the results of this study uncovered a functional interplay between RanBPM and ATM which suggests that RanBPM is involved in the regulation of the DNA damage response.

## 3. Functions

RanBPM has been implicated in the regulation of multiple pathways and cellular processes, but because it only consists of protein–protein interaction domains and is part of a large complex, it has been hypothesized to be a scaffolding protein. It has been shown to be associated with many protein partners and has been suggested to integrate different signalling pathways, implicating it in a variety of cellular functions both in the cytoplasm and in the nucleus [11,29,37,38] (table 1). Those functions include regulation of apoptosis, transcription, cell adhesion, migration and morphology.

**Table 1.** List of proteins that have been demonstrated to interact with RanBPM (n.d., not determined).

protein	interacting region of RanBPM	reference
*proteins that PTM RanBPM*		
ATM	n.d.	[36]
CDK11^p46^	SPRY domain	[33]
PLK1	n.d.	[34]
USP11	SPRY domain	[31]
*apoptosis*		
HIPK2	n.d.	[39]
p75NTR	n.d.	[40]
CDK11^p46^	SPRY domain	[33]
p73	aa 132–406	[41]
*transcription regulation*		
AR	SPRY domain	[22]
GR	SPRY domain	[22]
TR	C-terminal region	[42]
TAF4	C-terminal region	[30]
Rta	SPRY domain	[43]
Zta	SPRY domain	[44]
Mirk	n.d.	[45]
LFA-1	C-terminal region	[29]
TrkA	SPRY domain	[46]
TRAF6	SPRY domain	[47]
*cell adhesion, morphology and migration*		
LFA-1	C-terminal region	[29]
PlexinA1	N-terminal region	[48]
Muskelin	multiple domains	[49]
MET	SPRY domain	[23]
BLT2	SPRY domain	[50]
*cancer*		
c-Raf	n.d.	[51]
p73	aa 132–406	[41]
Mgl1	SPRY domain	[52]
HDAC6	LisH and CTLH domains	[53]
*Alzheimer's disease*		
APP	SPRY and LisH domains	[54]
LRP	SPRY and LisH domains	[54]
BACE1	SPRY and LisH domains	[54]
*development*		
MVH	n.d.	[55]
hSMP	n.d.	[56]
CITK	n.d.	[57]
YPEL5	SPRY domain	[58]
Cend1	SPRY, LisH and CTLH domains	[59]
Dyrk1A	n.d.	[45]
PlexinA1	N term	[48]
L1	N-terminal region and SPRY domain	[37]
p42^IP4^/Centaurin α-1	SPRY domain	[60]
TrkB	n.d.	[61]
TAF4	C-terminal region	[30]
FMRP	CRA domain	[13]
Calbindin D_28 K_	aa 401–407	[62]
mGlu receptors	aa 403–614	[63]
Obscurin	aa 108–729	[64]
Titin	n.d.	[64]
c-Kit	SPRY domain	[65]
*RNA processing*		
FMRP	CRA domain	[13]
SF3B3	n.d.	[66]
HNRNPM	n.d.	[66]
PABPC1	n.d.	[66]
PABPC2	n.d.	[66]
XPO5	n.d.	[67]
*CTLH complex*		
ARMC8α and β	n.d.	[27]
RMND5A	n.d.	[27]
MAEA	n.d.	[27]
Twa1	n.d.	[26]
Muskelin	n.d.	[26]
*others*		
α-tubulin	n.d.	[4]
Psoriasin	n.d.	[68]
IpaC	n.d.	[69]
PBGD	n.d.	[70]
Axl	SPRY domain	[71]
CD39	aa 1–148	[72]
Dectin 1E	SPRY domain	[73]
CBS	n.d.	[74]
RanBP10	LisH and CTLH domains	[21]
Ubc9	n.d.	[43]
Cav3.1	n.d.	[75]
MOP	n.d.	[76]
AChE	n.d.	[77]
PKCγ and δ	n.d.	[78]
Dopamine receptor D1	n.d.	[78]
COPS5 (Jab1)	SPRY and LisH domains	[79]
AICD	aa 136–460	[80]
Tip60	aa 136–460	[80]
TBRI	SPRY	[81]
STC2	n.d.	[82]
CCDC55	n.d.	[83]

### 3.1. Role in apoptosis

We identified a pro-apoptotic role for RanBPM through modulation of some members of the B-cell lymphoma 2 (Bcl-2) family of proteins, which are known to control the activation of the intrinsic apoptotic pathway [84]. Firstly, we found that ectopic expression of RanBPM resulted in increased caspase activation [5]. This effect appeared to be mediated by the CRA domain as deletion of this domain severely reduced caspase activation. Conversely, overexpression of a RanBPM SPRY domain deletion mutant resulted in increased caspase activation, suggesting that the SPRY domain restrains RanBPM's pro-apoptotic function. In addition, downregulation of RanBPM resulted in decreased apoptotic activation and increased cell survival following IR treatment [5]. Similarly, knockdown of RanBPM in gastric cancer cells resulted in increased cell survival after exposure to two different chemotherapeutic agents [85]. Further investigations showed that RanBPM exerts its pro-apoptotic function through a regulation of the intrinsic apoptotic pathway. Stable RanBPM downregulated cells showed increased protein levels of anti-apoptotic Bcl-2 family members Bcl-2 and Bcl-X_L_ and, following IR treatment, decreased mitochondrial localization of Bax, a pro-apoptotic Bcl-2 family member responsible for mitochondrial permeabilization and cytochrome-c release, a central step in the activation of apoptosis through the intrinsic pathway [5,86]. A later study confirmed that overexpression of RanBPM resulted in decreased Bcl-2 protein levels and increased Bax oligomerization [87]. This study also demonstrated that cells overexpressing RanBPM had increased mitochondrial membrane permeability and increased cytoplasmic cytochrome-c compared with control cells, demonstrating that RanBPM overexpression induces mitochondrial membrane depolarization resulting in the activation of apoptosis. Interestingly, neurons generated from RanBPM transgenic mice overexpressing RanBPM exhibited compromised mitochondrial function and increased amyloid-β (Aβ)-induced apoptosis [88,89]. Altogether, these studies suggest that RanBPM functions to promote apoptosis through a regulation of the intrinsic apoptotic pathway.

Interestingly, RanBPM has been suggested to have additional roles in other aspects of apoptotic activation through its interaction with various proteins functioning in regulating the apoptotic process. For example, RanBPM is able to bind to homeodomain-interacting protein kinase 2 (HIPK2), which has been suggested to promote apoptosis through its interaction with the death receptor tumour necrosis factor (TNF) receptor type 1-associated death domain protein [39]. RanBPM has been shown to interact with the intracellular death domain of p75 neurotrophin receptor (p75NTR), which is part of the TNF receptor superfamily [40]. Numerous adapter proteins have been shown to bind to the cytoplasmic domain of p75NTR and promote apoptosis, therefore, RanBPM's interaction with p75NTR death domain led to the suggestion that RanBPM could function to modulate p75NTR-induced apoptosis [40]. RanBPM also interacts with a caspase-processed fragment of CDK11^p110^, CDK11^p46^, which is involved in apoptotic signalling and overexpression of which can induce apoptosis [33]. These studies, however, did not investigate the functional outcome of these interactions; therefore, how RanBPM influences the function of these factors in the apoptotic process is unclear. Finally, RanBPM was shown to stabilize pro-apoptotic transcription factor p73 through inhibition of its ubiquitination and subsequent degradation [41]. RanBPM overexpression was also found to upregulate p73 mRNA [87], indicating that it stabilizes p73 at both the transcription and protein level. Again, how these regulations are achieved remains unknown, but collectively these reports indicate that RanBPM may regulate apoptosis at various steps in the apoptotic cascade activation.

### 3.2. Transcription regulation

As RanBPM partly localizes to the nucleus and has been shown to associate with chromatin, it is possible that RanBPM could function in transcription regulation [30]. Microarray analysis of stable control and RanBPM shRNA cells revealed global gene expression changes upon RanBPM downregulation [86]. Analysis of the transcription factor binding sites within the promoters of these genes identified homeobox A5 (HOXA5), forkhead box (FOX) and high mobility group proteins as being over-represented, suggesting that RanBPM's regulation of the transcriptome may occur through an interaction with members of these families of transcription factors. Indeed, RanBPM has been implicated in transcriptional regulation through its interaction with a number of transcriptional regulators. RanBPM directly interacts with steroid receptors and positively regulates their transcriptional activity in a ligand-dependent manner. This has been demonstrated for the androgen receptor (AR), the glucocorticoid receptor (GR) as well as the thyroid receptor (TR) [22,42,90]. RanBPM has been shown to interact with TATA box binding protein-associated factor 4 (TAF4), a subunit of transcription factor II D (TFIID) which functions as a transcriptional co-activator for several classes of transcription factors [30]. In the context of the Epstein-Barr virus (EBV) lytic cycle activation, RanBPM increased the transcriptional activity of both Rta and Zta through differential regulation of sumolyation of these proteins. Rta and Zta are transcription factors that activate the expression of key EBV lytic genes activating the EBV lytic cascade. RanBPM also mediates their interaction resulting in synergistic activation of their transcriptional activity [43,44].

RanBPM was shown to associate with minibrain-related kinase (Mirk) and inhibit its kinase activity as well as its transactivator activity [45]. RanBPM has also been shown to regulate the transcriptional activation of the activator protein 1 transcription factor complex, through its synergistic association with lymphocyte function-associated antigen-1 (LFA-1), a membrane integrin receptor [29]. RanBPM was reported to negatively regulate nerve growth factor stimulated nuclear factor of activated T cells (NFAT)-dependent transcription through its association with the tropomyosin-related kinase A (TrkA) receptor [46]. RanBPM also negatively regulated TNF receptor-associated factor 6 (TRAF6) E3 ligase activity, leading to decreased nuclear factor kappa B (NF-κB) signalling [47]. Collectively, these findings demonstrate that RanBPM regulates transcription directly, through interaction with transcription factors, and indirectly, acting as a signalling molecule upstream of transcriptional regulation.

### 3.3. Roles in adhesion, morphology and migration

Through its interaction with different proteins, RanBPM has been implicated to have a role in cell adhesion, morphology and migration. An early study showed that RanBPM associates with LFA-1, an integrin receptor with a role in cell adhesion [29]. Integrins mediate the adhesive interactions between cells or between cells and the extracellular matrix [91]. They act as signalling receptors transmitting information about extracellular environment inside the cells to affect the cell behaviour. Overexpression of RanBPM was shown to inhibit cell adhesion and delay cell spreading, and accordingly RanBPM knockdown promoted adhesion and spreading [92]. RanBPM inhibited the localization of cell adhesion proteins talin and vinculin at focal adhesions, thereby inhibiting focal adhesion assembly. In addition, RanBPM accelerated the endocytotic internalization of β1-integrin resulting in reduced levels at the cell surface, and again these effects were reversed when RanBPM was knocked down [92]. Similarly, RanBPM was shown to inhibit cell adhesion and cell morphology within the female germline stem cell niche in *Drosophila.* However, in the follicle RanBPM was a positive regulator of adhesion [93]. RanBPM associates with the PlexinA1 receptor, which is essential for the regulation of cell morphology [48]. In the presence of PlexinA1, overexpression of RanBPM resulted in cell contraction. In addition, RanBPM associates with Muskelin and knockdown of either Muskelin or RanBPM results in altered cell morphology including enlarged cell perimeter and altered actin distributions [49].

RanBPM was found to both negatively and positively affect cell motility. Through its interaction with MET proto-oncogene, receptor tyrosine kinase (MET), RanBPM activated the extracellular signal regulated kinase (ERK) pathway, stimulating cell motility [23]. However, this study employed a RanBPM–GFP fusion construct and the addition of a large fluorescent group could potentially have affected RanBPM function. As RanBPM was shown to dimerize [94], it is possible that the GFP tag could interfere with dimerization, therefore having adverse effects on RanBPM function. Alternatively, RanBPM was found to inhibit chemotactic migration by associating with leukotriene B4 receptor 2 (BLT2) [50]. Downregulation of RanBPM resulted in increased chemotactic migration, whereas overexpression of RanBPM was found to inhibit migration demonstrated by transwell migration assays. Our studies also demonstrated that cells with stable downregulation of RanBPM had increased migration compared with control cells in a wound healing scratch assay, indicating that RanBPM functions to inhibit cell migration [51]. Recently, a study performed in gastric cancer cells provided additional evidence that downregulation of RanBPM causes decreased cell adhesion and increased cell motility [85]. Taken together, RanBPM has been shown to function in inhibiting cell adhesion, altering cell morphology and inhibiting cell migration.

These cellular functions of RanBPM also suggested a much broader role for RanBPM in cancer, development and AD, implying that RanBPM has important functions in both normal and pathological development.

## 4. RanBPM in cancer

Several studies have suggested that RanBPM functions to prevent cell transformation through restricting the activation of several signalling pathways that promote tumourigenesis [5,29,38,41,50–52,81,86,95]. Work in our laboratory has suggested a tumour suppressor role for RanBPM through an inhibition of ERK signalling. RanBPM was found to interact with c-Raf and downregulate c-Raf protein levels. This is turn downregulates anti-apoptotic Bcl-2 family proteins at both the transcriptional and protein level [5,51]. Accordingly, cells with downregulated RanBPM were able to evade the activation of apoptosis, a common characteristic of cancer cells [5]. Downregulation of RanBPM resulted in loss of growth factor dependence as RanBPM shRNA cells were shown to continue to survive and proliferate in the absence of growth serum and also resulted in increased cell migration and cell proliferation, indicating that downregulation of RanBPM promotes cell transformation [51]. In addition, microarray analyses showed that one-third of differentially expressed genes between control and RanBPM shRNA are associated with cancer [86]. Pathways affected by downregulation of RanBPM included ERK, Wnt, Notch and phosphoinositide-3 kinase (PI3K)/Akt pathways. These pathways regulate cell cycle progression, cell proliferation, cell growth, differentiation, migration, adhesion and cell survival and are commonly found deregulated in cancer [96–99]. In the context of gastric cancer, downregulation of RanBPM also resulted in increased cell proliferation and cell motility [85].

There are several examples of interactions between RanBPM and proteins implicated in cancer development. As previously mentioned, RanBPM was shown to upregulate the mRNA levels of the pro-apoptotic transcription factor p73, thereby promoting the activation of apoptosis [87]. It was also shown to stabilize its protein expression by preventing ubiquitination and subsequent degradation [41]. Similarly, RanBPM was able to prevent the ubiquitination and subsequent degradation of the mammalian homologue of *Drosophila* tumour suppressor protein lethal giant larvae (Mgl1), by mediating its interaction with USP11. This resulted in prolonged half-life and enhanced the tumour suppressor properties of Mgl1, as Mgl1 was shown to inhibit cell migration and cell proliferation [52,95]. RanBPM was identified as an essential component of aggresome formation in response to both proteasomal inhibition and double-strand break-induced DNA damage [53]. This study identified that RanBPM, through its LisH and CTLH domains, associates with histone deacetylase 6 (HDAC6), a cytoplasmic deacetylase that is required for aggresome formation, and that RanBPM inhibits HDAC6 deacetylase activity [53]. HDAC6 is known to promote many aspects of cancer development such as cell transformation and angiogenesis, and therefore HDAC6 has become a major target for therapeutic interventions to treat cancer [100,101]. The inhibitory effect of RanBPM on HDAC6 activity could therefore represent a major role for RanBPM in preventing cancer development, although the specific effects of RanBPM on HDAC6 cancer-promoting activities remain to be further explored.

**Figure 2.**
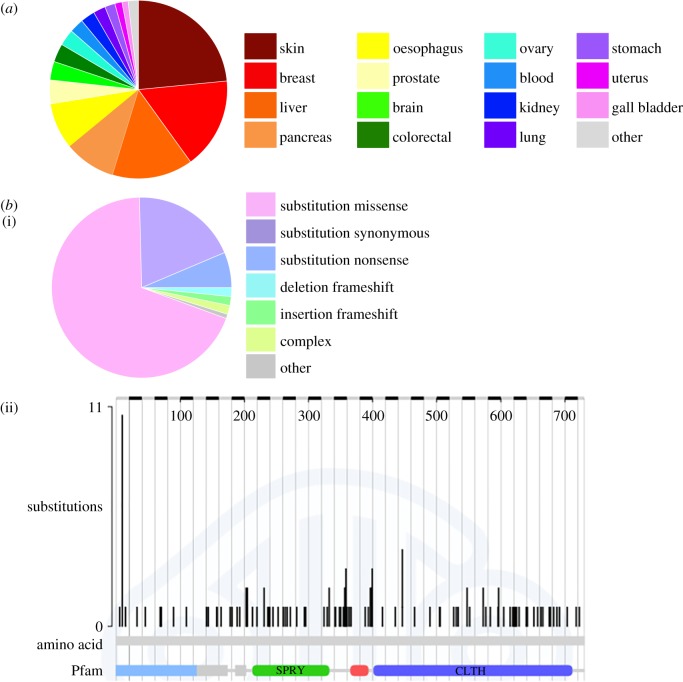
(*a*) Tissue distribution of RanBPM mutations retrieved from the ICGC database. Figure adapted from the ICGC database [103]. (*b*) (i) Relative abundance of substitution mutations in RanBPM. (ii) Histogram indicating the position of somatic mutations in RanBPM. Figures adapted from the COSMIC database [104]. Frequency of RanBPM mutations in the COSMIC database is 0.43%.

A study of gastric cancers identified lower levels of RanBPM transcripts in tumours with distant metastases than in tumours without distant metastases, implying that loss of RanBPM expression may promote the development of metastases [85]. In addition, RanBPM expression was decreased or altered in lung, kidney and breast primary tumours, raising the possibility that in these cancer types RanBPM functions as a tumour suppressor [29]. Furthermore, the Candidate Cancer Gene Database identified RanBPM as a cancer driver gene mutation using analysis of transposon mutagenesis in mice, with the highest rank of common insertion sites being liver cancer [102]. Interestingly, the International Cancer Genome Consortium (ICGC) identifies skin, breast and liver as the primary sites harbouring the majority of RanBPM mutations [103] (figure 2*a*). The Catalogue of Somatic Mutations in Cancer (COSMIC) database indicates that, while the incidence of RanBPM mutations are relatively low (0.43%), the mutations are mostly nucleotide substitutions and are distributed throughout the entire gene [104] (figure 2*b*). It is also worth noting that a large-scale analysis of SNPs associated with early breast cancer cases identified 41 new SNPs, one of them being upstream of RanBPM [105].

While overall the data points towards RanBPM acting as a tumour suppressor, there is some evidence that suggests it may also have a cancer-promoting role. For example, an early paper determined that RanBPM interacts with the receptor tyrosine kinase MET and found that a GFP–RanBPM fusion protein stimulates Ras activation and cell migration [23]. Also, RanBPM was identified as a negative regulator of minibrain-related kinase (Mirk)/Dyrk1B, an inhibitor of cell migration [45]. Notably, the COSMIC database shows that RanBPM transcripts are more often overexpressed than downregulated in tumour samples [104]. The apparent opposing actions of RanBPM on tumourigenesis are likely to be context-dependent. Determining the actions of RanBPM in the context of cell type and other mutations is a necessary step to elucidating the complex role RanBPM has during cancer development.

Overall, the published experimental evidence points towards a tumour suppressor role for RanBPM. Therefore, further understanding of its regulation and function is crucial in order to be able to exploit its ability to inhibit cancer progression in a therapeutic setting.

## 5. RanBPM in Alzheimer's disease

RanBPM has been shown to play a pathogenic role in AD as it acts as a scaffold bringing together amyloid precursor protein (APP), low-density lipoprotein receptor-related protein (LRP) and β-site APP-cleaving enzyme 1 (BACE1) [54]. This complex promotes the cleavage of APP to Aβ, the toxic fragment resulting in Aβ pathological plaques, a major hallmark of AD, at the expense of the non-pathogenic APP processing by α-secretase cleavage [106]. Knockdown of RanBPM resulted in a decreased secretion of Aβ and RanBPM overexpression decreased the amount of APP at the cell surface due to increased internalization, which is necessary for its pathogenic cleavage [54]. An *in vivo* mouse study mirrored these results, further confirming that RanBPM overexpression significantly increased the generation of Aβ in an AD mouse model [107]. A comparison of human AD brains to those of age-matched controls found increased levels of a RanBPM proteolytic fragment, N60 RanBPM [94]. This N60 fragment was actually found to be more potent than full-length RanBPM in enhancing β-secretase processing of APP. It remains unclear how this proteolytic fragment is generated, but it has been shown to be dependent on cell density, as cells at low density express higher levels of the N60 fragment compared with cells at a higher confluency [94].

RanBPM also has been implicated in the regulation of synapses. Overexpression of RanBPM in transgenic mice resulted in reduced levels of pre- and post-synaptic proteins, suggesting a role for RanBPM in the cognitive impairment which accompanies AD [107,108]. In a mouse model of AD, overexpression of RanBPM resulted in increased levels of neuroinflammation and increased synaptic functional impairment due to reduction of synaptic proteins and loss of dendritic intersections and spines [89,109–112]. Increased RanBPM expression also exacerbated the deficiencies in spatial learning and memory displayed by AD mice [109].

RanBPM overexpression was shown to promote activated cofilin, which normally functions as a regulator of actin dynamics [89,113]. Cofilin activation occurs through positive regulation of slingshot homologue 1 (SSH1) expression, which dephosphorylates and activates cofilin [114]. Translocation of cofilin to the mitochondria results in mitochondrial dysfunction and promotes apoptosis [113,115]. Neurons generated from AD mice overexpressing RanBPM demonstrated deficits in the ability to clear Ca^2+^ to the mitochondria, resulting in increased reactive oxygen species production contributing to decreased synaptic function [88]. These cells also exhibited increased levels of Aβ-induced apoptosis dependent on cofilin expression. Overexpression of RanBPM has been shown to induce apoptosis through the intrinsic mitochondria-mediated pathway in neurons, therefore likely contributing to neurodegeneration in AD [87]. Conversely, an AD mouse model with reduced expression of RanBPM showed decreased levels of Aβ accumulation, reduced neuroinflammation and mitigated the loss of post-synaptic protein expression compared to AD mice expressing normal RanBPM levels, suggesting that RanBPM mediates several aspects of Aß neurotoxic effects [113].

Taken together these results indicate that RanBPM has a pathological role in the major hallmarks contributing to AD: Aβ production and decreased synaptic function and density, neuronal death contributing to neurological defects, and can even accelerate disease pathology, indicating that RanBPM may be a therapeutic target for AD [110].

## 6. RanBPM in development

Two different groups have generated RanBPM knockout (KO) mice. Both groups found Mendelian proportion of RanBPM KO embryos *in utero*, indicating that RanBPM is not required for embryonic development [116,117]. The first group, Puveral *et al.* [116], found that less than the expected number of KO pups were born and many died immediately after birth. Palavicini *et al*. [117] reported that very few KO mice survived past 24 h, citing a failure to latch and suckle milk resulting in a lack of nourishment and a loss of bodily homeostasis in the absence of liquid as cause of death. Both studies reported, however, that surviving KO mice were significantly smaller than their wild-type litter mates [116,117].

Puveral *et al*. [116] further noted that both male and female RanBPM KO mice were sterile, and closer examination showed that RanBPM is essential for spermatogenesis and oogenesis. Both genders were found to have a meiotic defect occurring around the late pachytene/diplotene stages, therefore suggesting the presence of a common mechanism requiring RanBPM function occurring in both genders at the end of prophase I. RanBPM was also shown to play a role in spermatogenesis in the new world common marmoset [118]. RanBPM was previously suggested to function in spermatogenesis, as it is able to associate with mouse vasa homologue (MVH), a vasa protein, which plays an essential role in the development of the male germ cell [20,55,116]. Recently, it was identified that RanBPM associates with and positively regulates the levels of c-Kit, a tyrosine kinase receptor expression of which is essential for haematopoietic stem cells and for the production of male and female gametes [65,119]. Interestingly, loss of c-Kit function in mice result in defective spermatogonia proliferation at the pre-meiotic stages, similarly to what was observed in RanBPM KO mice [65]. In addition, RanBPM was shown to regulate mRNA splicing imperative for normal spermatogenesis and male fertility, as a conditional RanBPM KO mouse showed that many transcripts in the testes were aberrantly spliced contributing to sterility in male mice [66]. Its association with human sperm membrane protein-1 has also implicated RanBPM to function in spermiogenesis [56]. Finally, RanBPM was found to be an essential gene in *Drosophila* where it has two isoforms, long and short RanBPM. Both isoforms contain the central domains, the long isoform also contains an unstructured N-terminal region that is glutamine-rich. The long isoform of RanBPM plays an important role in the niche development of the female ovaries and the short isoform negatively regulates the organization of the germ stem cell niche [93].

RanBPM was suggested to have a role in the regulation of mitosis through its interaction with PLK1, citron kinase (CITK), yippee like 5 (YPEL5), cycle exit and neuronal differentiation 1 (Cend1) and dual specificity tyrosine-phosphorylation regulated kinase 1B (Dyrk1B) [34,57–59]. PLK1 is crucial for M-phase progression having a role in centrosome maturation and bipolar spindle formation [120]. However, the functional relevance of its association with RanBPM remains to be determined [34]. CITK functions downstream of PLK1 and plays a role in mitosis and cytokinesis and downregulation of RanBPM results in an increase in mitotic cells due to slower entry into cytokinesis, suggesting that RanBPM functions to promote progression of mitosis by promoting entry into cytokinesis [57]. YPEL5 is localized with the mitotic machinery throughout the cell cycle, and is involved in cell cycle progression and knockdown of YPEL5 suppressed growth rates and prolonged G1, G2 and M phases [58]. Nonetheless, the functional consequences of the association of RanBPM and YPEL5 on cell cycle progression were not investigated [58]. RanBPM was also shown to be involved in influencing the cellular processes controlling cycle progression versus differentiation of neuronal precursors through interactions with BM88/Cend1 and Dyrk1B [59]. Differential interaction between RanBPM and BM88/Cend1 and Dyrk1B was reported to affect the stabilization of the protein levels of cyclin D1 which controls the balance between cellular proliferation and differentiation, therefore implicating RanBPM in the regulation of the progression of the cell cycle. Altogether, these studies suggest that RanBPM has the potential to regulate various steps of the mitotic pathway. However, the mechanisms of these regulations remain to be fully elucidated.

Palavicini *et al.* [117] went on to characterize a severe deficit in brain development in their RanBPM KO mice. KO mice displayed reduced brain volumes and enlarged lateral ventricles. They attributed the failure to suckle milk resulting in death to abnormalities in brain development including defects in somatosensory systems, neuromuscular or craniofacial development. They suggest that RanBPM acts as a scaffold within a complex that regulates neurite outgrowth and neuronal migration, which are imperative for proper brain growth and development. Previous studies have implicated a role for RanBPM in neurite outgrowth because of its interaction with PlexinA1 receptor [48], L1 receptor [37], p42^IP4^/centaurin α-1 [60], TrkB [61], and Dyrk1B and Cend1 [59]. Through association with TAF4, RanBPM was shown to have a role in neural stem cell differentiation and seems to play a role in the initiation of neurite processes [30]. Also, RanBPM interacts with fragile X mental retardation protein (FMRP), transcriptional silencing of which results in fragile X syndrome, the most common form of inherited mental retardation [13]. RanBPM function has been shown to be required in the nervous system of *Drosophila* for proper larval feeding behaviour, response to light and for coordinated locomotion. RanBPM was found highly expressed within the mushroom body of *Drosophila* and re-expression of RanBPM in neurons within the mushroom body was sufficient to rescue all behavioural phenotypes of RanBPM mutant larvae [121]. Other examples of RanBPM's role in the neuronal system include its interaction with calbindin D_28 K_, which has a role as a calcium buffer and calcium sensor important in neuronal function [62], and its interaction with several metabotropic glutamate receptors (mGlu), particularly mGlu2, suggesting a role for RanBPM as a scaffold in the neuronal system at synaptic sites [63]. RanBPM was shown to interact with obscurin and titin suggesting that RanBPM is involved in the development of Z-discs in skeletal muscle. However, the function of RanBPM in this process remains to be fully elucidated [64]. Recent investigation of the RanBPM KO mice uncovered that compared to wild-type, RanBPM KO mice show a significant decrease in bone marrow progenitor cells, suggesting a role for RanBPM in normal haematopoietic development [65]. Finally, microarray analyses performed by our laboratory showed that genes involved in embryonic, cellular and tissue development were over-represented among those differentially expressed between control and RanBPM shRNA cells [86]. In particular, RanBPM downregulation affected several pathways involved in development and cancer, such as the Wnt and Notch pathways, and the top biological functions identified included haematological and reproductive system development and function. These analyses suggest that RanBPM expression affects genes and pathways that regulate developmental programmes and are linked to tumourigenesis when deregulated.

Overall, while much remains to be investigated regarding the exact function of RanBPM in many of the aforementioned processes where RanBPM has been implicated, RanBPM appears to have critical functions during development, particularly brain development, haematopoiesis and gametogenesis.

## 7. CTLH complex

During the initial characterization of RanBPM, gel filtration analysis demonstrated it was part of a 670 kDa complex [2]. Later, two-hybrid-associated protein 1 with RanBPM (Twa1), Muskelin, armadillo repeat containing 8 (ARMC8) α and β, required for meiotic nuclear division homologue A (Rmnd5A), and macrophage erythroblast attacher (MAEA) were identified to associate with RanBPM by mass spectrometry in RanBPM immunoprecipitates and/or yeast two hybrid screen using RanBPM as bait [26,27]. These proteins exhibited identical sedimentation behaviours in a sucrose gradient assay, confirming that they were all part of the same complex. The complex was named the CTLH (C-terminal to LisH) complex, because, with the exception of ARMC8, a striking feature is the shared presence of LisH and CTLH domains amongst the complex members [27]. Additional evidence for the existence of this complex in mammals comes from several proteomics studies. The CTLH complex was identified as a co-regulator complex for nuclear receptors [122], further implying a role for the complex in the regulation of steroid receptor transcriptional activation shown in previous studies [22,42,90]. The CTLH complex was found to be part of the Axin interactome, although the role of the complex on Axin function was not investigated [123]. In addition, two different studies identified the CTLH complex as a component of the cilium [124,125]. A homologous complex exists in *Arabidopsis thaliana* [24] and *Saccharomyces cerevisiae* [3,126], except that the LisH and CTLH domain-containing protein WD repeat domain 26 (WDR26 or GID7 in yeast) is found instead of Muskelin, which is not encoded in these organisms.

### 7.1. Yeast GID complex activity

The CTLH complex is conserved in yeast where it is called the glucose-induced degradation-deficient (GID) complex. When yeast cells are grown in ethanol and then switched to a glucose-rich medium, the GID complex becomes activated and mediates ubiquitination resulting in proteasomal degradation of fructose-1,6-bisphophatase (FBPase), a key enzyme required for gluconeogenesis [3,126,127]. This is a necessary step in a process called catabolite inactivation which allows yeast cells to favour glycolysis instead of gluconeogenesis. Each GID protein is necessary for FBPase ubiquitination and degradation [3,126]. Two central GID complex subunits, GID2 (Rmnd5A) and GID9 (MAEA), contain RING finger domains [3,128]. The presence of a RING finger domain is a characteristic of E3 ubiquitin ligases and RING domains function to mediate the transfer of ubiquitin to specific substrates [129]. Ubiquitin ligase activity has been demonstrated for GID2 *in vitro* [3], while attempts to show GID9 E3 ligase activity were unsuccessful [128]. Interestingly, a mutation of a cysteine residue in the GID9 RING domain impairs FBPase ubiquitination but does affect heterodimerization with GID2 [128]. This suggests that while only GID2 has intrinsic ubiquitin ligase activity, a fully intact GID9 RING domain is needed for FBPase ubiquitination. An understanding of the cooperation of the two RING domains in the ubiquitination activity of this complex is an interesting area for further investigation. Like GID2, Rmnd5A orthologues in *Xenopus laevis* and *Lotus japonicas* have *in vitro* ubiquitin ligase activity [130,131], but activity in higher organisms has yet to be demonstrated.

Interestingly, one GID complex member, GID4, is absent in glucose-starving conditions but is immediately synthesized after yeast cells are switched to a glucose-rich media, coinciding with degradation of FBPase [3]. Recent work has revealed that GID4 is the recognition component of the GID complex, specifically recognizing the N-terminal proline of FBPase, and two other gluconeogenic enzymes that are also ubiquitinated by the GID complex, isocitrate lyase 1 (Icl1) and malate dehydrogenase 2 (Mdh2) [132]. This indicates that the synthesis of GID4 upon glucose replacement and its subsequent association to the GID complex drives recruitment of gluconeogenic enzymes to the GID complex leading to their ubiquitination and proteasomal degradation. Shortly after FBPase is eliminated, GID4 levels also deplete indicating a negative feedback loop [3]. Importantly, this work on GID4 recognition of FBPase demonstrated a novel N-end rule pathway leading to proteasomal degradation, termed the Pro/N-end rule pathway [132].

The human orthologue of GID4, C17orf39, was not identified in the mammalian CTLH complex, but possibly, like in yeast, its association is signal-dependent. It remains to be determined whether the Pro/N-end recognition by GID4 is a conserved feature in mammals, but regardless, GID4/C17orf39 represents a strong candidate as a protein responsible for targeting specific substrates to this putative E3 ubiquitin ligase complex. The human orthologues of FBPase and Mdh2 (Icl1 does not have a human orthologue) do not have N-terminal prolines, suggesting that either they have evolved to be regulated by an alternative mechanism or human GID4/C17orf39 recognizes a different sequence motif in these proteins in mammals. Identifying targets of the CTLH complex will provide mechanistic insights into the recognition of substrates by mammalian GID4/C17orf39 or other members of the complex.

### 7.2. Vacuole-dependent degradation of FBPase

When yeast have been starved in ethanol for 3 days, glucose re-feeding triggers the degradation of FBPase through an alternative pathway, the vacuole-dependent degradation pathway, which has similarities to both to the macro-autophagy pathway and the lysosomal pathway in mammalian cells [133,134]. Vid30/Gid1 with Vid24 are required for the vacuole degradation of FBPase and play an important role promoting the association of Vid vesicles and actin patches thus integrating the Vid and endocytic pathways [133,135].

### 7.3. Mammalian CTLH complex

**Figure 3.**
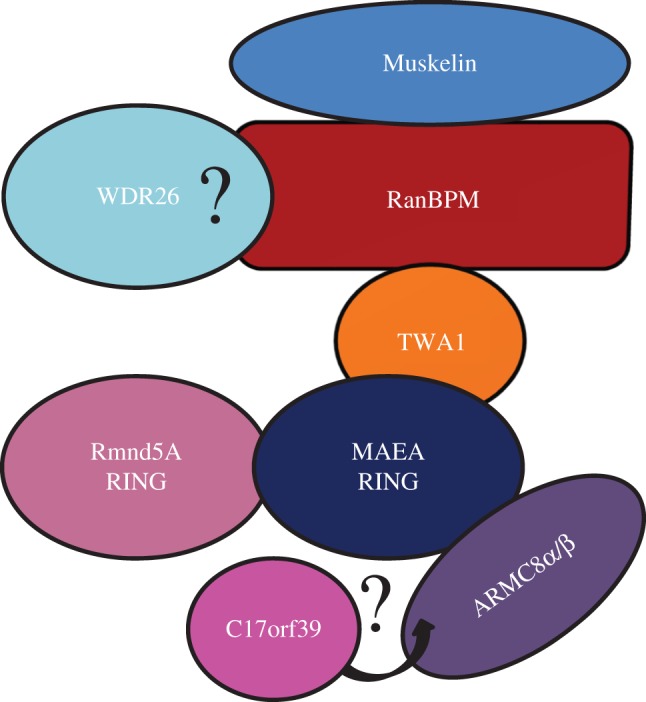
Proposed model of the topology of the mammalian CTLH complex. The model is adapted from that of the yeast GID complex described in Menssen *et al*. [127] as the topology of the mammalian complex has not been elucidated. The stoichiometry of the complex is not known. WDR26 association with the complex remains to be formally demonstrated.

Immunoprecipitation experiments in yeast ΔGID mutant strains have provided a model of the architecture of the GID complex [127]: GID7 (WDR26) is directly bound to GID1 (RanBPM), GID1 and GID8 (TWA1) directly interact and form the core of the complex, the GID2 (Rmnd5A)/GID9 (MAEA) heterodimer associates with GID1 through GID8, while GID4 and GID5 (ARMC8) are on the periphery and connect to the complex via GID9. Because of the evolutionary conservation of the complex [28], a model for the mammalian version of this arrangement can be deduced (figure 3). One notable difference from the yeast complex is that GID7 and GID4 were not identified in the mammalian complex [27]. Instead, it has been speculated that Muskelin replaces GID7 [28].

RanBPM is the most well-studied mammalian protein in the CTLH complex, whereas only a few studies have been conducted on other complex members. Through head-to-tail binding and dimerization via its LisH domains, the cytoplasmic protein Muskelin forms a tetramer and has been implicated in the process of internalizing GABA receptors from the cell surface in neurons [136,137]. MAEA (also known as erythroblast macrophage protein, EMP) null embryos die perinatally and show a defect in erythroblast enucleation and macrophage maturation [138]. Studies on ARMC8α in mammalian cell lines show that it positively regulates α-catenin degradation and, when overexpressed, promotes interactions of hepatocyte growth factor-regulated tyrosine kinase substrate protein (HRS) with ubiquitinated proteins [139,140]. Suppression of Rmnd5A expression in *Xenopus laevis* impairs embryonic forebrain development, although the molecular basis for this phenotype was not investigated [130]. WDR26, a putative member of the CTLH complex, has been reported to be involved in the regulation of several signalling pathways including the phosphatidylinositol 3-kinase (PI3K) pathway and Gβγ-mediated G protein signalling [141,142]. As previously mentioned, a recent study revealed that the CTLH complex associates with Axin, a scaffolding protein involved in β-catenin ubiquitination and degradation [123]. WDR26 was identified in the complex and was subsequently found to have an inhibitory effect on β-catenin levels [143]. Interestingly, RanBPM has been linked to the regulation of β-catenin levels in *Drosophila* [93] and ARMC8 was previously found to associate with β-catenin [139], further implicating the complex in the regulation of WNT signalling.

Many of the studies referenced above have studied RanBPM in isolation; however, whether RanBPM functions alone or as a part of the CTLH to fulfil the many roles attributed to RanBPM remains to be identified. As the substrates targeted by the CTLH complex remain to be investigated, it would be of interest to evaluate if the proteins identified to interact or associate with RanBPM are substrates of the CTLH complex.

## Data accessibility

This article has no additional data.
